# Perioperative outcomes of HoLEP, ThuLEP, and TURP in patients with prostate cancer: results from the GRAND study

**DOI:** 10.1038/s41391-025-00980-x

**Published:** 2025-05-19

**Authors:** Nikolaos Pyrgidis, Gerald Bastian Schulz, Philipp Weinhold, Michael Atzler, Leo Federico Stadelmeier, Iason Papadopoulos, Christian Stief, Julian Marcon, Patrick Keller

**Affiliations:** Department of Urology, University Hospital LMU, Munich, Germany

**Keywords:** Diseases, Prostatic diseases

## Abstract

**Background:**

Limited data exist on the role of holmium laser enucleation of the prostate (HoLEP), thulium laser enucleation of the prostate (ThuLEP), and transurethral resection of the prostate (TURP) in patients with prostate cancer (PCa). We aimed to analyze their perioperative outcomes and trends.

**Materials and methods:**

The German Nationwide Inpatient Data (GRAND) registry was used to identify male patients diagnosed with PCa who underwent HoLEP, ThuLEP, or TURP between 2005 and 2022. Multivariable regression analyses were performed to compare perioperative morbidity and mortality.

**Results:**

A total of 221,768 procedures in patients with PCa were performed: 8160 HoLEP, 2285 ThuLEP, and 211,323 TURP. Although TURP remains the predominant technique, the use of HoLEP and ThuLEP has increased significantly in recent years, representing 17% of all cases by 2022. Perioperative outcomes were worse for TURP, with higher transfusion (8.8%) and ICU admission rates (1.7%) compared to HoLEP and ThuLEP (both 2.5% and ≤1.2%, respectively). Postoperative urinary retention and incontinence rates were also lower for laser enucleation techniques than TURP. In patients with PCa undergoing TURP, perioperative outcomes were worse compared to those without PCa, while outcomes for HoLEP and ThuLEP were comparable regardless of PCa status.

**Conclusion:**

HoLEP and ThuLEP offer improved perioperative outcomes compared to TURP in patients with PCa.

## Introduction

Lower urinary tract symptoms (LUTS) due to benign prostatic hyperplasia (BPH) are highly prevalent among elderly men [1]. Transurethral resection of the prostate (TURP) is the gold-standard surgical treatment for BPH [2]. Laser enucleation with holmium laser enucleation of the prostate (HoLEP) or thulium laser enucleation of the prostate (ThuLEP) is also considered among the first-line surgical treatment modalities [3] Incidental prostate cancer (PCa) is highly prevalent among patients undergoing TURP, HoLEP, and ThuLEP and can be found in up to 20% of all specimens [4, 5]. Moreover, TURP, HoLEP, or ThuLEP may become necessary in patients with low-risk PCa and severe LUTS, patients with LUTS and intermediate- or high-risk PCa before radiotherapy (to prevent radiotherapy-induced exacerbation of LUTS), or patients with LUTS due to locally advanced or metastatic PCa [6].

TURP remains widely used, mainly when access to laser technologies is limited or surgeon expertise in laser enucleation is lacking [7]. However, the adoption of HoLEP and ThuLEP has grown significantly in recent years, supported by evidence demonstrating the benefits of laser enucleation in reducing complications and managing larger prostates [8]. Despite these advancements, TURP is still considered the standard surgical procedure for managing LUTS in patients with PCa [9]. That said, accumulating evidence suggests that HoLEP and ThuLEP may be safe and effective alternatives for such patients [10]. In particular, the available studies indicate that laser enucleation is feasible not only in patients with localized PCa but also in those with locally advanced or metastatic PCa [11]. Still, to date, limited data comparing the trends and perioperative outcomes of TURP, HoLEP, and ThuLEP in patients with PCa exist.

Understanding these differences is crucial for optimizing surgical decision-making and improving patient outcomes [12]. In this context, we aimed to provide a comprehensive analysis of surgical trends and perioperative outcomes among patients with PCa undergoing TURP, HoLEP, or ThuLEP over the last two decades.

## Methods

### GeRmAn Nationwide inpatient Data (GRAND)

This study utilized data from the German Nationwide Inpatient Data (GRAND) registry, which is maintained by the Federal Bureau of Statistics in Wiesbaden, Germany, and contains anonymized patient-level information on all inpatient nationwide hospitalizations from 2005 to 2022. Military, psychiatric, and forensic cases are excluded. Our team worked exclusively with aggregated summary data provided by the Research Data Center and did not have access to individual patient records. Access to the anonymized dataset was granted following the necessary approvals (LMU - 4710-2022) and this study did not require any ethical review or patient informed consent according to the German regulations.

Since the introduction of the Diagnosis Related Groups (DRG) payment system in 2005, German hospitals have submitted patient data on hospital diagnoses, perioperative complications, and surgical procedures to the Institute for the Hospital Remuneration System. Diagnoses and complications are coded using the International Classification of Diseases, 10th Revision, German Modification (ICD-10-GM), while surgical procedures are coded using the German Procedure Classification (OPS). The German Institute for Medical Documentation and Information ensures standardized coding practices across the country.

### Data source

We identified male patients diagnosed with PCa (ICD-10-GM code: C61) who underwent HoLEP (OPS code: 5-601.60), ThuLEP (OPS code: 5-601.61), or TURP (OPS code: 5-601). The dataset included information on demographics, comorbidities, year of surgery, and perioperative outcomes. Comorbidities were identified using ICD-10-GM codes.

The primary objective of this study was to compare patients with PCa versus no PCa undergoing HoLEP, ThuLEP, and TURP in terms of perioperative morbidity. Secondary outcomes included the comparison of patients with PCa versus no PCa undergoing HoLEP, ThuLEP, and TURP in terms of perioperative morbidity [sepsis, postoperative incontinence, urinary retention, transfusion rates, acute kidney disease, and intensive care unit (ICU) admissions, as well as the length of hospital stay]. Further secondary analyses examined the trends in the use of HoLEP, ThuLEP, and TURP for PCa, as well as the perioperative outcomes of HoLEP and ThuLEP versus TURP in patients with PCa.

### Statistical analysis

Multivariable logistic regression models were employed to assess the association between surgical approach and binary outcomes and multivariable linear regression models were used to analyze the length of hospital stay as a continuous outcome. Models were adjusted for patient age, comorbidities, and year of surgery to control for confounding factors. Continuous variables were summarized as medians with interquartile ranges (IQR), while categorical variables were reported as frequencies and proportions. Odds ratios (ORs) with 95% confidence intervals (CIs) were calculated, and statistical significance was set at a *p*-value threshold of <0.05. The Research Data Center performed the filtering of the study population with SAS and the statistical analysis with R scripts developed by our team (source: Research Data Center, DRG Statistics 2005–2022).

## Results

### Baseline characteristics

Between 2005 and 2022, 221,768 procedures were performed in Germany for patients with PCa. Of them, 211,323 (95%) included TURP, 8160 (3.7%) HoLEP, and 2285 (1.3%) ThuLEP. Still, HoLEP and ThuLEP started to be coded separately in 2012 and 2018, respectively. Patients undergoing TURP were slightly older than those undergoing HoLEP and ThuLEP, but the comorbidities among the three groups were comparable. The baseline characteristics of the included patients are summarized in Table 1.

**Table 1:** Baseline characteristics of patients with prostate cancer undergoing HoLEP, ThuLEP, and TURP.

Characteristic	HoLEP, *n* = 8160	ThuLEP, *n* = 2285	TURP, *n* = 211,323
Age (years)	75 (69–80)	75 (69–80)	76 (70–81)
Diabetes	1475 (18%)	407 (18%)	41,353 (20%)
Chronic heart failure	315 (3.9%)	79 (3.5%)	13,748 (6.5%)
COPD	443 (5.4%)	145 (6.3%)	15,086 (7.1%)
Chronic kidney disease	548 (6.7%)	155 (6.8%)	26,516 (13%)
Hypertension	10,554 (51%)	1256 (55%)	118,194 (56%)
Obesity	299 (3.7%)	115 (5.0%)	11,477 (5.4%)
Year of surgery			
2012	259 (3.2%)	0	12,104 (5.7%)
2013	345 (4.2%)	0	11,376 (5.4%)
2014	372 (4.6%)	0	11,448 (5.4%)
2015	426 (5.2%)	0	11,537 (5.5%)
2016	611 (7.5%)	0	11,479 (5.4%)
2017	694 (8.5%)	0	11,690 (5.5%)
2018	735 (9.0%)	387 (17%)	11,680 (5.5%)
2019	975 (12%)	426 (19%)	11,561 (5.5%)
2020	1068 (13%)	403 (18%)	10,274 (4.9%)
2021	1266 (16%)	464 (20%)	10,229 (4.8%)
2022	1409 (17%)	605 (26%)	10,194 (4.8%)

Variables are presented as median (interquartile range) or frequencies with proportions.

*COPD* chronic obstructive pulmonary disease, *HoLEP* holmium laser enucleation of the prostate, *ThuLEP* thulium laser enucleation of the prostate, *TURP* transurethral resection of the prostate.

**Fig. 1:**
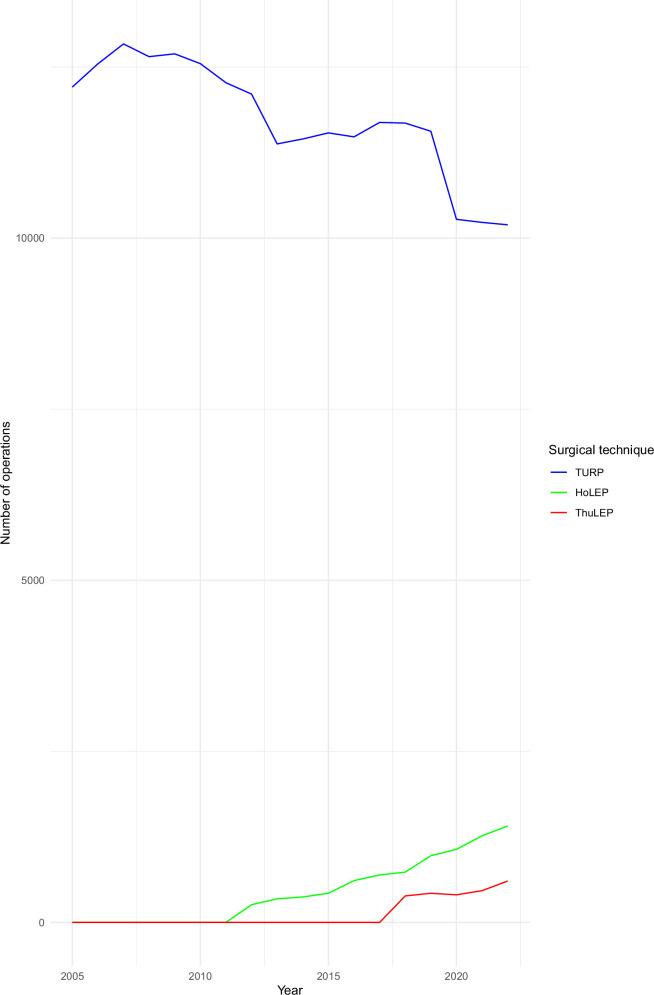
The annual trends of HoLEP, ThuLEP, and TURP in patients with prostate cancer in Germany. HoLEP holmium laser enucleation of the prostate, ThuLEP thulium laser enucleation of the prostate, TURP transurethral resection of the prostate.

The number of PCa cases performed annually with TURP is slightly decreasing. On the other hand, the use of HoLEP and ThuLEP has increased significantly, particularly in the last few years. By 2022, HoLEP and ThuLEP represented about 17% of all PCa cases. Figure 1 illustrates the annual trends of the three surgical approaches.

### Perioperative outcomes of HoLEP, ThuLEP, and TURP

Among patients with PCa, those undergoing TURP presented worse perioperative outcomes compared to those undergoing HoLEP or ThuLEP. In particular, transfusions and ICU admissions were more frequent in patients undergoing TURP (8.8% and 1.7%, respectively) compared to HoLEP (2.5% and 1.2%, respectively) and ThuLEP (2.5% and 0.8%, respectively). Similarly, in patients with PCa undergoing laser enucleation, all major perioperative complications were better and the length of hospital stay was shorter compared to TURP. Accordingly, the postoperative urinary retention and incontinence rates were better after HoLEP (9.8% and 4.6%, respectively) and ThuLEP (9% and 3%, respectively) versus TURP (17% and 4.8%, respectively). All perioperative outcomes are detailed in Table 2.

**Table 2:** Perioperative outcomes of patients with prostate cancer undergoing HoLEP, ThuLEP, and TURP.

Characteristic	HoLEP, *n* = 8160	ThuLEP, *n* = 2285	TURP, *n* = 211,323
Mortality	14 (0.2%)	Less than 3 cases	1816 (0.9%)
ICU admission	100 (1.2%)	19 (0.8%)	3658 (1.7%)
Transfusion	204 (2.5%)	58 (2.5%)	18,508 (8.8%)
Sepsis	50 (0.6%)	22 (1.0%)	2026 (1.0%)
Acute kidney disease	121 (1.5%)	35 (1.5%)	7203 (3.4%)
Postoperative incontinence	372 (4.6%)	68 (3%)	10,086 (4.8%)
Urinary retention	798 (9.8%)	206 (9%)	36,869 (17%)
Length of hospital stay (days)	4 (3–5)	3 (3–5)	6 (5–9)

Variables are presented as median (interquartile range) or frequencies with proportions.

*HoLEP* holmium laser enucleation of the prostate, *ICU* intensive care unit, *ThuLEP* thulium laser enucleation of the prostate, *TURP* transurethral resection of the prostate.

In the multivariable analysis, comparing the perioperative outcomes of patients with PCa versus no PCa undergoing TURP, patients with PCa presented worse perioperative outcomes. Except for ICU admissions, all the evaluated perioperative outcomes (including mortality, transfusion, sepsis, acute kidney disease, postoperative incontinence, urinary retention, and length of hospital stay) were statistically significantly worse in patients with PCa versus no PCa. On the contrary, patients with PCa undergoing HoLEP or ThuLEP presented comparable perioperative outcomes compared to those with no PCa undergoing HoLEP or ThuLEP. The comparisons for patients with PCa versus no PCa for the three techniques are available in Table 3.

**Table 3:** Multivariable linear and logistic regression analysis for the perioperative outcomes of patients with no PCa versus PCa undergoing HoLEP, ThuLEP, and TURP.

Outcome	HoLEP		ThuLEP		TURP	
	Proportion (no PCa vs PCa)	Estimate (95% CI), *p*-value	Proportion (no PCa vs PCa)	Estimate (95% CI), *p*-value	Proportion (no PCa vs PCa)	Estimate (95% CI), *p*-value
Mortality	<0.1% vs 0.2%	1.39 (0.73, 2.48), 0.3	<0.1% vs <0.1%	0.31 (0.02, 1.55), 0.3	0.2% vs 0.9%	**2.52 (2.37, 2.69)**, <**0.001**
ICU admission	0.9% vs 1.2%	1.14 (0.91, 1.41), 0.2	0.8% vs 0.8%	0.99 (0.58, 1.60), >0.9	1.5% vs 1.7%	1.01 (0.97, 1.05), 0.5
Transfusion	1.7% vs 2.5%	1.10 (0.94, 1.28), 0.2	2.3% vs 2.5%	0.86 (0.64, 1.15), 0.3	3.6% vs 8.8%	**2.03 (1.99, 2.07)**, <**0.001**
Sepsis	0.5% vs 0.6%	1.14 (0.83, 1.54), 0.4	0.5% vs 1%	1.54 (0.92, 2.47), 0.08	0.7% vs 1%	**1.16 (1.10, 1.22)**, <**0.001**
Acute kidney disease	0.9% vs 1.5%	**1.31 (1.06, 1.59), 0.01**	1.3% vs 1.5%	0.90 (0.61, 1.28), 0.6	1.1% vs 3.4%	**2.39 (2.32, 2.47)**, <**0.001**
Postoperative incontinence	4.5% vs 4.6%	0.93 (0.83, 1.04), 0.2	2.5% vs 3%	1.11 (0.84, 1.44), 0.4	3.4% vs 4.8%	**1.28 (1.25, 1.31)**, <**0.001**
Urinary retention	6.5% vs 7.3%	1.04 (0.95, 1.14), 0.3	6.7% vs 9%	**1.23 (1.04, 1.44), 0.01**	9.1% vs 13%	**1.36 (1.34, 1.38)**, <**0.001**
Length of hospital stay	4d vs 4d	0.03 (−0.05, 0.10), 0.5	3d vs 3d	−0.15 (−0.30, 0.01), 0.06	6d vs 6d	**0.78 (0.75, 0.81)**, <**0.001**

All models are adjusted for age, diabetes, chronic renal failure, hypertension, and obesity. The bold cells indicate statistically significant *p*-values.

*CI* confidence interval, *HoLEP* holmium laser enucleation of the prostate, *ICU* intensive care unit, *PCa* prostate cancer, *ThuLEP* thulium laser enucleation of the prostate, *TURP* transurethral resection of the prostate.

## Discussion

The present nationwide analysis from Germany provides comprehensive data on the perioperative outcomes and trends of TURP, HoLEP, and ThuLEP in patients with PCa. Our findings demonstrate that laser enucleation techniques offer significantly better perioperative outcomes compared to TURP, including lower rates of transfusions, ICU admissions, and postoperative urinary retentions. Accordingly, patients with PCa undergoing HoLEP or ThuLEP display similar perioperative outcomes compared to those without PCa undergoing HoLEP or ThuLEP. Notably, the use of HoLEP and ThuLEP has increased steadily in recent years, comprising approximately 17% of all PCa cases by 2022, while TURP cases have slightly declined. Still, TURP is the operation of choice for managing LUTS in patients with PCa.

It is important to emphasize that HoLEP, ThuLEP, and TURP can be utilized in various clinical scenarios of PCa, ranging from incidental, low-risk cases to those with locally advanced or metastatic disease. Laser enucleation is based on identifying and following the surgical plane, which might not be feasible in patients with infiltrating PCa [13]. Based on the previous notion, available evidence indicates that the enucleation time, as well as the surgical performance (g/min), are worse in patients with PCa compared to patients with no PCa [14]. Nevertheless, it seems that the latter does not negatively affect the short- and long-term outcomes of laser enucleation. Indeed, previous studies indicate that laser enucleation offers adequate symptom improvement and a good safety profile, even in patients with locally advanced PCa [10, 14].

On the contrary, our findings suggest that TURP is associated with worse perioperative outcomes in patients with PCa compared to both patients with PCa undergoing laser enucleation as well as to those without PCa undergoing TURP [15]. Indeed, TURP in patients with PCa was associated with the highest rates of perioperative complications. The latter might be explained by the fact that TURP is selected in cases where laser enucleation is unfeasible or at least technically challenging, such as in patients with locally advanced PCa, emergency operations, or multiple comorbidities [16]. In an attempt to overcome this selection bias, we present a high-volume cohort with multiple patient-level analyses, comparing both PCa versus no PCa patients, as well as TURP versus laser enucleation. In line with our findings, previous studies suggest that TURP is associated with worse outcomes in patients with known PCa [17, 18]. Conversely, the favorable perioperative outcomes of HoLEP and ThuLEP underline the clinical advantages of laser enucleation, especially for patients with PCa, who are often at higher risk of adverse events due to comorbidities or disease progression [19].

It should be stressed that our findings demonstrate a swift towards the use of HoLEP and ThuLEP in patients with PCa, which reflects the global trends of BPH surgery [20]. Of note, it seems that an important amount of urologists in Germany performing HoLEP and ThuLEP opt for laser enucleation even in the case of PCa. This shift suggests a growing preference for minimally invasive procedures that may maximize patient safety and recovery while maintaining efficacy even in challenging cases [21]. However, despite the increasing use of laser enucleation, TURP continues to be the mainstay for BPH surgery due to its established role in clinical practice, broader availability, and familiarity among surgeons [22]. Moreover, the use of laser enucleation may be limited by the higher initial costs associated with laser equipment, as well as by the steep learning curve for surgeons, especially in patients with PCa [23].

Our findings underscore important clinical implications. First, laser enucleation should be considered as a viable surgical option in patients with PCa [24]. The safety profile of laser techniques may reduce postoperative morbidity, facilitating faster recovery also for challenging cases [25]. Therefore, our results support a paradigm shift in surgical management of LUTS in PCa patients. Future studies should assess long-term functional and oncologic outcomes and evaluate implementation strategies for broader adoption of laser enucleation [26]. Importantly, integrating laser enucleation into standard practice will require structured training programs to overcome the steep learning curve [27]. Moreover, healthcare systems should consider investing in laser technology infrastructure, especially in high-volume centers, to maximize accessibility and patient benefit [28].

This study represents, to the best of our knowledge, the largest analysis of the perioperative outcomes and trends of TURP, HoLEP, and ThuLEP in patients with PCa. However, several important limitations should be noted. First of all, the retrospective design and reliance on billing data may introduce potential inaccuracies due to coding errors or misclassificationsm as well as due to underreporting or overreporting of some outcomes. Additionally, important clinical details such as tumor stage (incidental or advanced PCa), complete oncological status (i.e., Gleason score, tumor size, TNM classification), operative time, patient laboratory findings (including PSA levels), and detailed patient comorbidity profiles (including ASA score or Charlson Comorbidity Index) were not available for analysis. Accordingly, histopathological findings and prior PCa treatments were missing. Based on the previous notion, no subgroup analyses (e.g. advanced versus not advanced PCa) could be performed. Due to the structure of the GRAND registry, we were unable to identify or exclude repeat surgeries. As such, it is possible that a small number of patients who underwent more than one intervention over the study period may be included more than once. Still, in an attempt to overcome these limitations, we performed a holistic analysis comparing PCa versus no PCa patients, as well as TURP versus laser enucleation. Of note, the absence of long-term data on functional outcomes, recurrence rates, and patient quality of life further limits the conclusions of this study. Lastly, as the findings are specific to the German healthcare system, caution is warranted in extrapolating these results to countries with different healthcare systems and surgical practices.

Our analysis highlights the increasing adoption of laser enucleation techniques, such as HoLEP and ThuLEP. Patients with PCa undergoing laser enucleation display similar perioperative outcomes compared to those without PCa. On the contrary, TURP in patients with PCa is associated with worse perioperative outcomes compared to patients with PCa undergoing HoLEP or ThuLEP as well as compared to patients without PCa undergoing TURP. However, these findings should be interpreted with caution, as TURP is the preferred surgical approach in specific clinical contexts where laser enucleation may not be feasible or indicated, such as in patients with locally advanced disease and severe LUTS. Each surgical approach has unique advantages and limitations that must be carefully weighed based on patient-specific factors and surgeon expertise to ensure optimal clinical decision-making.

## Data Availability

All data generated or analyzed during this study are included in this article. Further inquiries can be directed to the corresponding author.
